# Bisoprolol and/or hyperoxic breathing do not reduce hyperventilation in pulmonary arterial hypertension patients

**DOI:** 10.1177/20458940211057890

**Published:** 2021-12-01

**Authors:** Eva L. Peters, Jasmijn S.J.A van Campen, Herman Groepenhoff, Frances S. de Man, Anton Vonk Noordegraaf, Harm J. Bogaard

**Affiliations:** ^1^ Department of Pulmonary Medicine Amsterdam Cardiovascular Sciences, Amsterdam UMC Vrije Universiteit Amsterdam Amsterdam The Netherlands; ^2^ Department of Physiology Amsterdam Cardiovascular Sciences, Amsterdam UMC Vrije Universiteit Amsterdam Amsterdam The Netherlands

**Keywords:** pulmonary hypertension, hyperventilation, sympathetic nervous system, beta blocker, autonomic imbalance

## Abstract

Hyperventilation is common in pulmonary arterial hypertension and may be related to autonomic imbalance. Patients underwent exercise testing and hyperoxic breathing before and after bisoprolol treatment. We found that neither beta blocker treatment nor hyperoxic breathing in patients reduced hyperventilation at rest and during exercise, although it reduced heart rate.

## Research letter

Pulmonary arterial hypertension (PAH) is a rare but severe disease, characterized by obstructive remodelling of pulmonary arteries and leading to right ventricular failure.^1^ PAH is incurable, and treatment options are limited. The main symptoms of PAH are (exertional) dyspnoea, impaired exercise performance and reduced quality of life.^2^

PAH patients often present with an increased minute volume (VE) at rest (i.e. hyperventilation) and a steeper slope of ventilation relative to carbon dioxide production (VE/VCO_2_) during exercise.^3^, ^4^, ^5^ Hyperventilation in PAH is related to disease severity^3^, ^5^, ^6^ and is associated with hypocapnia, a strong and independent prognostic value for survival.^7^ In addition, it may further exaggerate the feelings of dyspnoea. The mechanisms underlying hyperventilation in PAH are poorly understood, but include increased dead space ventilation and, possibly, autonomic imbalance and hypoxemia, leading to alveolar hyperventilation.^6^, ^8^, ^9^, ^10^

It has been shown previously that four months of carvedilol treatment improves ventilatory efficiency in chronic heart failure patients.^11^ In addition, patients taking beta blockers showed to have lower minute ventilation (VE) both at rest and during exercise compared to patients not on beta blockers.^12^ However, we are not aware of studies on the effect of long‐term beta blocker use on ventilation in PAH patients. Yet, acute hyperoxic breathing showed to increase ventilatory efficiency in PAH patients during, but not at the start of exercise.^13^

Thus, we hypothesized that lowering sympathetic activity and/or acute hyperoxic breathing will reduce hyperventilation. We took advantage of the bisoprolol trial in PAH^14^ to investigate whether long‐term beta blocker treatment or acute hyperoxic breathing, known to reduce sympathetic activity,^10^ lowers ventilation in PAH patients.

Informed consent was obtained from all patients and a Data Safety Monitoring Board (DSMB) was appointed. The trial was registered at clinicaltrials.gov before recruitment was initiated (Clinicaltrials.gov NCT01246037, EudraCT 2010‐020424‐21). The study design and in‐ and exclusion criteria, and the cardiopulmonary exercise testing (CPET) protocol have been described in detail elsewhere.^14^ In short, all idiopathic PAH patients above 18 years of age and in New York Heart Association (NYHA) class II and III were screened for eligibility. Patients received six months of bisoprolol and placebo treatment in a cross‐over manner, in random order. At baseline, before cross‐over and at the end of the study, patients underwent CPET, arterial blood gas (ABG) analysis and ventilatory measurements. ABG was taken from the radial artery after at least 10 min of supine rest. Ventilatory measurements were performed in absolute resting condition, in a supine position in a quiet room. After breathing room‐air for at least 10 min, VE, tidal volume (VT), respiratory rate (RR) and end‐tidal CO_2_ (P_ET_CO_2_) were measured breath‐by‐breath using a metabolic cart (Vmax Encore 21‐1, Yorba Linda, USA) and analysed as 20 s averages. Oxygen saturation (SaO_2_) was measured by pulse‐oximetry (9600, Nonin, Plymouth, USA) and heart rate by electrocardiography (Eagle 4000, Marquette). After 10 min, the inspired oxygen fraction (FiO_2_) was changed. Measurements were performed with an FiO_2_ of 21% and 40%, in random order, both for 10 min. Blinding codes were broken on the last day of the third admission or at early termination of the study. However, all data were analysed in a blinded fashion. Statistics were performed using GraphPad Prism 7. Two‐way repeated measures ANOVA was used to test for the effects of bisoprolol at baseline and after treatment, and to test the effects of hyperoxic breathing both after placebo and bisoprolol treatment. *p* < 0.05 was considered statistically significant. Continuous variables are presented as mean  ±  standard deviation (SD).

Eighteen patients were enrolled into the study from February 2011 until January 2014, of whom 17 underwent CPET and ventilatory measurements at baseline. Fifteen out of 17 patients received placebo for the full six months during the placebo arm of the study. In the bisoprolol arm of this study, 16 patients received bisoprolol for six months.^14^ As such, a complete paired set of data is available from 15 patients.

As a reflection of our general PAH population, NYHA class II and III were equally represented, mean age was 46 ± 14 years and there was a strong female predominance (only one male patient was included). Average mean arterial pressure (mPAP) was 48 ± 11 mmHg and 6‐minute walking distance 468 ± 84 m. Results of CPET, ABG and ventilatory measurements at baseline and after placebo and bisoprolol treatment are shown in Table 1. Baseline measurements showed a high minute ventilation and a low P_ET_CO_2_ and PaCO_2_. There were no signs of metabolic alkalosis.

The reached dosage of bisoprolol (4.5  ±  3.3 mg on average) was associated with a reduction in heart rate of 12 bpm (*p* = 0.004) at rest in normoxia, suggesting decreased sympathetic nerve activity. However, minute ventilation, tidal volume or respiratory rate were unchanged. No changes in P_ET_CO_2_ or PaCO_2_ were observed, indicating an unchanged ventilation and perfusion. At baseline, VE/VCO_2_ slope was elevated (43.3 ± 10.8), indicating reduced ventilatory efficiency during exercise. Bisoprolol did not reduce heart rate at the start of CPET or at maximum exercise. Neither ventilatory efficiency, VE_peak_, RR_peak_, VT_peak_ or P_ET_CO_2_ were changed after bisoprolol treatment.

**Table 1:** Effects of bisoprolol and hyperoxic breathing on ventilation at rest and during exercise.

(Mean ± SD)	Baseline (n = 17)	Placebo (n = 16)	Bisoprolol (n = 16)	Effect of bisoprolol (*p*)	Effect of hyperoxia (*p*)	Interaction bisoprolol×hyperoxia (*p*)
CPET						
Work (watt)	66 ± 30	59 ± 34	67 ± 31	*0.2914*		
VO_2_AT (l/min)	0.70 ± 0.20	0.64 ± 0.24	0.65 ± 0.20	*0.5194*		
VO_2_max (l/min)	1.01 ± 0.34	0.95 ± 0.41	0.98 ± 0.34	*0.5200*		
VO_2_/kg (ml/kg/min)	15.2 ± 4.6	13.8 ± 5.2	14.6 ± 4.5	*0.9337*		
HR start (bpm)	80 ± 14	76 ± 13	74 ± 18	*0.8204*		
HR max (bpm)	138 ± 21	124 ± 26	123 ± 27	*0.9279*		
O_2_pulse (ml/beat)	7.2 ± 1.7	7.6 ± 2.7	7.9 ± 2.1	*0.6477*		
VE_peak_ (l/min)	54.2 ± 24.0	48.1 ± 28.0	49.4 ± 16.7	*0.9444*		
RR_peak_ (min^–1^)	38 ± 18	35 ± 20	36 ± 10	*0.8462*		
VT_peak_ (l)	1.4 ± 0.4	1.4 ± 0.4	1.4 ± 0.3	*0.2105*		
VE/VCO_2_ slope	42.4 ± 11.0	42.1 ± 11.5	39.8 ± 11.0	*0.8425*		
ABG						
PaCO_2_ (mmHg)	32.3 ± 4.7	32.2 ± 0.8	32.8 ± 3.1	*0.5737*		
PaO_2_ (mmHg)	73.5 ± 21.5	67.2 ± 13.0	71.3 ± 15.8	*>0.9999*		
VentFiO_2_ 21%						
VT (l)	0.8 ± 0.2	0.7 ± 0.2	0.8 ± 0.2	0.0149*		
VE (l/min)	11.0 ± 3.0	10.9 ± 2.1	10.7 ± 2.8	*0.1751*		
RR (breaths/min)	14.5 ± 3.1	15.8 ± 3.0	15.1 ± 4.1	*0.2784*		
P_ET_CO_2_ (kPa)	3.6 ± 0.6	3.7 ± 0.6	3.7 ± 0.4	*0.4822*		
SaO_2_ (%)	93.0 ± 5.7	92.4 ± 5.2	92.0 ± 7.0	*0.3458*		
HR (bpm)	78 ± 12	78 ± 13	66 ± 13	<0.0001*		
Vent FiO_2_ 40%						
VT (l)	0.8 ± 0.2	0.8 ± 0.2	0.8 ± 0.2	0.0149*	*0.4726*	*0.1634*
VE (l/min)	11.2 ± 2.7	11.0 ± 2.4	11.3 ± 2.6	*0.1751*	*0.2742*	*0.1518*
RR (breaths/ min)	14.7 ± 3.4	16.3 ± 2.9	15.4 ± 4.2	*0.2784*	*0.6032*	*0.4689*
P_ET_CO_2_ (kPa)	3.6 ± 0.6	3.7 ± 0.6	3.7 ± 0.5	*0.4822*	*0.2418*	*0.7034*
SaO_2_ (%)	96.9 ± 4.4	96.1 ± 4.9	96.1 ± 6.4	*0.3458*	0.0012*	*0.8132*
HR (bpm)	74 ± 12	75 ± 13	64 ± 13	<0.0001*	0.0044*	*0.2906*

*p*‐values for the main effects of bisoprolol and hyperoxia, as well as the interaction‐effect, are given in italic numbers. *p < 0.05.AT: anaerobic threshold; HR: heart rate; VE: total minute ventilation; RR: respiratory rate; VT: tidal volume; P_ET_CO_2_: end‐tidal partial pressure of CO_2_; SaO_2_: oxygen saturation; FiO_2_: inspired fraction of oxygen.

Ten minutes of hyperoxic breathing with FiO_2_ = 40% significantly increased SaO_2_ with 3.9% (*p* = 0.0012) and decreased heart rate with 3.6 bpm (*p* = 0.0044). However, no effect was found on ventilation as reflected by VE, RR, VT and P_ET_CO_2_. In addition, no interaction‐effect between bisoprolol treatment and hyperoxic breathing at rest was found for any of the outcome variables, indicating no cumulative or opposing effects of bisoprolol and hyperoxic breathing.

Collectively, our results show that ventilation at rest is not affected by bisoprolol or hyperoxic breathing, either alone or in combination. Furthermore, bisoprolol did not improve ventilatory inefficiency during exercise. The reductions in heart rate following bisoprolol treatment and hyperoxic breathing strongly suggest decreased SNS activity.

Limitations of the present study are the small sample size and the wide range of bisoprolol doses in our patients. We did not directly measure sympathetic nerve activity or chemoreceptor activity, and therefore cannot prove nor exclude the role of the sympathetic nervous system in hyperventilation in PAH. The lack of effect in the current study may thus be related to the relatively low dose of bisoprolol, the type of beta blocker,^15^ or the level and short duration of hyperoxia. However, oxygen has also central effects on respiratory centres in the brain stem, possibly via ROS signalling, which causes long‐lasting increases in ventilation.^16^ Whilst the use of this effect is unknown, it may have masked the initial reduction in ventilation due to peripheral chemoreceptor silencing. Alternatively, other factors may drive ventilation in PAH, including mechanical forces in the pulmonary circulation, low work rate lactic acidosis, hyperkalaemia and altered central regulation of breathing.^5^, ^17^, ^18^ Given the strong prognostic value of VE/VCO_2_ and hypocapnia, unravelling the aetiology of hyperventilation in PAH may help to improve treatment and thereby enhance survival and quality of life.

## Clinicaltrials.gov identifier

NCT01246037

## EudraCT

2010‐020424‐21

## Conflict of interest

The author(s) declare that there is no conflict of interest.

## Authors’ contribution

All authors made substantial contribution to the design, data acquisition, data analysis and interpretation of the data. All authors critically revised the article and provided final approval of the version for publication.

## Ethical approval

Written informed consent was obtained from all patients, and a Data Safety Monitoring Board (DSMB) was appointed. The trial was registered at clinicaltrials.gov before recruitment was initiated (Clinicaltrials.gov NCT01246037, EudraCT 2010‐020424‐21).

## Guarantor

HJB.

## Funding

The author(s) disclosed receipt of the following financial support for the research, authorship, and publication of this article: This research was financially supported by ZonMW (95110079). HJB, AVN and FSdM were supported by the Netherlands CardioVascular Research Initiative: The Dutch Heart Foundation, Dutch Federation of University Medical Centers, the Netherlands Organization for Health Research and Development, and the Royal Netherlands Academy of Sciences (CVON‐2012‐08 PHAEDRA, CVON‐2018‐29 PHAEDRA‐IMPACT, CVON‐2017‐10 Dolphin‐Genesis). HJB and AVN were supported by research grants from Actelion, GSK and Ferrer (Therabel). EP, AVN and FSdM were further supported by The Netherlands Organization for Scientific Research (NWO‐VICI: 918.16.610, NWO‐VIDI: 917.18.338).

## ORCID iDs

Eva L. Peters https://orcid.org/0000‐0001‐6846‐2154

Harm J. Bogaard https://orcid.org/0000‐0001‐5371‐0346

## Supporting information

Supplementary Material

Supplementary Material
