# Supplementary material for: Bisoprolol and/or hyperoxic breathing do not reduce hyperventilation in pulmonary arterial hypertension patients
Source: Pulm Circ. 2021 Dec 1;11(4):20458940211057890. doi: 10.1177/20458940211057890 (PMC8671678; doi:10.1177/20458940211057890)
Supplement: Supplementary file 2 — Supplementary Material [file PUL2-11-20458940211057890-s001.pdf]

# Bisoprolol and/or hyperoxic breathing do not reduce hyperventilation in pulmonary arterial hypertension patients

E.L. Peters[1,2*], J.S.J.A van Campen[1*], H. Groepenhoff[1], F.S. de Man[1], A. Vonk Noordegraaf[1], H.J. Bogaard[1]

[1] Pulmonary Medicine, Amsterdam Cardiovascular Sciences, Amsterdam UMC, Vrije Universiteit Amsterdam, Amsterdam, The Netherlands
[2] Department of Physiology, Amsterdam UMC, Vrije Universiteit Amsterdam, Amsterdam, The Netherlands

* authors contributed equally to this work

**Corresponding Author:** Prof. Harm Jan Bogaard, MD, PhD;
Department of Pulmonary Medicine
Amsterdam Cardiovascular Sciences
Amsterdam UMC, location VUmc
De Boelelaan 1117
1081 HV Amsterdam
The Netherlands
Tel: +31 20 44 4328
Fax: +31 20 44 4328
e-mail: hj.bogaard@amsterdamumc.nl

Clinicaltrials.gov identifier: NCT01246037
EudraCT: 2010-020424-21

Article type: research letter
Total number of pages: 9
Words: 1193 (excluding acknowledgements)
Figures/Tables: 1
References: 18
Keywords: Pulmonary Hypertension, Hyperventilation, sympathetic nervous system, beta blocker

**Abstract**

Hyperventilation is common in pulmonary arterial hypertension and may be related to autonomic imbalance. Patients underwent exercise testing and hyperoxic breathing before and after bisoprolol treatment. We found that neither beta blocker treatment, nor hyperoxic breathing in patients reduced hyperventilation at rest and during exercise, although it reduced heart rate.

**Research letter**

Pulmonary arterial hypertension (PAH) is a rare but severe disease, characterized by obstructive remodelling of pulmonary arteries and leading to right ventricular failure [1]. PAH is incurable and treatment options are limited. The main symptoms of PAH are (exertional) dyspnoea, impaired exercise performance and reduced quality of life [2].

PAH patients often present with an increased minute volume (VE) at rest (*i.e.* hyperventilation) and a steeper slope of ventilation relative to carbon dioxide production ($VE/VCO_2$) during exercise [3–5]. Hyperventilation in PAH is related to disease severity [3,5,6] and is associated with hypocapnia, a strong and independent prognostic value for survival [7]. In addition, it may further exaggerate the feelings of dyspnoea. The mechanisms underlying hyperventilation in PAH are poorly understood, but include increased dead space ventilation and, possibly, autonomic imbalance and hypoxemia leading to alveolar hyperventilation [6,8–10].

It has been shown previously that 4 months of carvedilol treatment improves ventilatory efficiency in chronic heart failure patients [11]. In addition, patients taking beta blockers showed to have lower minute ventilation (VE) both at rest and during exercise compared to patients not on beta blockers [12]. However, we are not aware of studies on the effect of long-term beta blocker use on ventilation in PAH patients. Yet, acute hyperoxic breathing showed to increase ventilatory efficiency in PAH patients during, but not at the start of exercise [13].

Thus, we hypothesized that lowering sympathetic activity and/or acute hyperoxic breathing will reduce hyperventilation. We took advantage of the bisoprolol trial in PAH [14] to investigate whether long-term beta blocker treatment or acute hyperoxic breathing, known to reduce sympathetic activity [10], lowers ventilation in PAH patients.

Informed consent was obtained from all patients and a Data Safety Monitoring Board (DSMB) was appointed. The trial was registered at clinicaltrials.gov before recruitment was initiated (Clinicaltrials.gov NCT01246037, EudraCT 2010-020424-21). The study design and in- and exclusion criteria and the cardiopulmonary exercise testing (CPET) protocol have been described in detail elsewhere [14]. In short, all idiopathic PAH patients above 18 years of age and in New York Heart Association (NYHA) class II and III were screened for eligibility. Patients received 6 months of bisoprolol and placebo treatment in a cross-over manner, in random order. At baseline, before cross-over, and at the end of the study patients underwent CPET, arterial blood gas (ABG) analysis and ventilatory measurements. ABG was taken from the radial artery after at least ten minutes of supine rest. Ventilatory measurements were performed in absolute resting condition, in a supine position in a quiet room. After breathing room-air for at least ten minutes, VE, tidal volume (VT), respiratory rate (RR) and end-tidal $CO_2$ ($P_{ET}CO_2$) were measured breath-by-breath using a metabolic cart (Vmax Encore 21-1, Yorba Linda, USA) and analysed as 20 seconds averages. Oxygen saturation ($SaO_2$) was measured by pulse-oximetry (9600, Nonin, Plymouth, USA) and heart rate by electrocardiography (Eagle 4000, Marquette). After ten minutes, the inspired oxygen fraction ($FiO_2$) was changed. Measurements were performed with an $FiO_2$ of 21% and 40%, in random order, both for 10 minutes. Blinding codes were broken on the last day of the third admission or at early termination of the study. However, all data were analysed in a blinded fashion. Statistics were performed using GraphPad Prism 7. Two-way repeated measures ANOVA was used to test for the effects of bisoprolol at baseline and after treatment, and to test the effects of hyperoxic breathing both after placebo and bisoprolol treatment. $p<0.05$ was considered statistically significant. Continuous variables are presented as mean ± standard deviation (SD).

18 patients were enrolled into the study from February 2011 until January 2014 of whom 17 underwent CPET and ventilatory measurements at baseline. 15 out of 17 patients received placebo for the full six months during the placebo arm of the study. In the bisoprolol arm of this study, 16 patients received bisoprolol for 6 months [14]. As such, a complete paired set of data is available from 15 patients.

As a reflection of our general PAH population, NYHA class II and III were equally represented, mean age was 46±14 years and there was a strong female predominance (only one male patient was included). Average mean arterial pressure (mPAP) was 48±11 mmHg and 6-minute walking distance 468±84m. Results of CPET, ABG and ventilatory measurements at baseline and after placebo and bisoprolol treatment are shown in table 1. Baseline measurements showed a high minute ventilation, and a low $PetCO_2$ and $PaCO_2$. There were no signs of metabolic alkalosis.

The reached dosage of bisoprolol (4.5 ± 3.3 mg on average) was associated with a reduction in heart rate of 12 bpm ($p$=0.004) at rest in normoxia, suggesting decreased sympathetic nerve activity. However, minute ventilation, tidal volume or respiratory rate were unchanged. No changes in $PetCO_2$ or $PaCO_2$, were observed, indicating an unchanged ventilation and perfusion. At baseline, $VE/VCO_2$ slope was elevated (43.3±10.8), indicating reduced ventilatory efficiency during exercise. Bisoprolol did not reduce heart rate at the start of CPET or at maximum exercise. Neither ventilatory efficiency, $VE_{peak}$, $RR_{peak}$, $VT_{peak}$ or $P_{ET}CO_2$ were changed after bisoprolol treatment.

Ten minutes of hyperoxic breathing with $FiO_2$=40% significantly increased $SaO_2$ with 3.9% ($p$ =0.0012) and decreased heart rate with 3.6 bpm ($p$ = 0.0044). However, no effect was found on ventilation as reflected by VE, RR, VT and $P_{ET}CO_2$. In addition, no interaction-effect between bisoprolol treatment and hyperoxic breathing at rest was found for any of the outcome variables, indicating no cumulative or opposing effects of bisoprolol and hyperoxic breathing.

Collectively, our results show that ventilation at rest is not affected by bisoprolol or hyperoxic breathing, either alone or in combination. Furthermore, bisoprolol did not improve ventilatory

inefficiency during exercise. The reductions in heart rate following bisoprolol treatment and hyperoxic breathing strongly suggest decreased SNS activity.

Limitations of the present study are the small sample size and the wide range of bisoprolol doses in our patients. We did not directly measure sympathetic nerve activity or chemoreceptor activity, and therefore cannot prove nor exclude the role of the sympathetic nervous system in hyperventilation in PAH. The lack of effect in the current study may thus be related to the relatively low dose of bisoprolol, the type of beta blocker [15], or the level and short duration of hyperoxia. However, oxygen has also central effects on respiratory centres in the brain stem, possibly via ROS signalling, which causes long-lasting increases in ventilation [16]. Whilst the use of this effect is unknown, it may have masked the initial reduction in ventilation due to peripheral chemoreceptor silencing. Alternatively, other factors may drive ventilation in PAH, including mechanical forces in the pulmonary circulation, low work rate lactic acidosis, hyperkalaemia and altered central regulation of breathing [5,17,18]. Given the strong prognostic value of $VE/VCO_2$ and hypocapnia, unravelling the aetiology of hyperventilation in PAH may help to improve treatment and thereby enhance survival and quality of life.

**Conflict of interest**

The authors declare that there is no conflict of interest

**Funding**

This research was financially supported by ZonMW [95110079]. HJB, AVN and FSdM were supported by the Netherlands CardioVascular Research Initiative: The Dutch Heart Foundation, Dutch Federation of University Medical Centers, the Netherlands Organization for Health Research and Development, and the Royal Netherlands Academy of Sciences [CVON-2012-08 PHAEDRA, CVON-2018-29 PHAEDRA-IMPACT, CVON-2017-10 Dolphin-Genesis]. HJB and AVN were supported by research grants from Actelion, GSK and Ferrer (Therabel). EP, AVN and FSdM were further supported by The Netherlands Organization for Scientific Research [NWO-VICI: 918.16.610, NWO-VIDI: 917.18.338].

**Ethical approval**

Informed consent was obtained from all patients and a Data Safety Monitoring Board (DSMB) was appointed. The trial was registered at clinicaltrials.gov before recruitment was initiated (Clinicaltrials.gov NCT01246037, EudraCT 2010-020424-21).

**Guarantor**

HJB is the guarantor for this article.

**Contributorship**

All authors contributed to manuscript preparation and provided final approval of the version for publication.

**Acknowledgements**

Not applicable

## References


1. Galiè N, Humbert M, Vachiery J-L, et al. 2015 ESC/EAS Guidelines for the diagnosis and treatment of pulmonary hypertension. *Eur Heart J* 2016; 12: 79–86.
2. Sajkov D, Petrovsky N, Palange P. Management of dyspnea in advanced pulmonary arterial hypertension. *Curr Opin Support Palliat Care* 2010; 4: 76–84.
3. Sun XG, Hansen JE, Oudiz RJ, et al. Exercise pathophysiology in patients with primary pulmonary hypertension. *Circulation* 2001; 104: 429–435.
4. Wensel R, Opitz CF, Anker SD, et al. Assessment of survival in patients with primary pulmonary hypertension: Importance of cardiopulmonary exercise testing. *Circulation* 2002; 106: 319–324.
5. Yasunobu Y, Oudiz RJ, Sun XG, et al. End-tidal Pco2 abnormality and exercise limitation in patients with primary pulmonary hypertension. *Chest* 2005; 127: 1637–1646.
6. Dimopoulos S, Anastasiou-Nana M, Katsaros F, et al. Impairment of autonomic nervous system activity in patients with pulmonary arterial hypertension: a case control study. *J Card Fail* 2009; 15: 882–889.
7. Hoeper MM, Pletz MW, Golpon H, et al. Prognostic value of blood gas analyses in patients with idiopathic pulmonary arterial hypertension. *Eur Respir J* 2007; 29: 944–950.
8. Naeije R, Van de Borne P. Clinical relevance of autonomic nervous system disturbances in pulmonary arterial hypertension. *Eur Respir J* 2009; 34: 792–794.
9. Farina S, Bruno N, Agalbato C, et al. Physiological insights of exercise hyperventilation in arterial and chronic thromboembolic pulmonary hypertension. *Int J Cardiol* 2018; 259: 178–182.
10. Velez-Roa S, Ciarka A, Najem B, et al. Increased sympathetic nerve activity in pulmonary artery hypertension. *Circulation* 2004; 110: 1308–1312.
11. Agostoni P, Guazzi M, Bussotti M, et al. Carvedilol reduces the inappropriate increase of ventilation during exercise in heart failure patients. *Chest* 2002; 122: 2062–2067.
12. Wolk R, Johnson BD, Somers VK, et al. Effects of β-blocker therapy on ventilatory responses to exercise in patients with heart failure. *J Card Fail* 2005; 11: 333–339.
13. Ulrich S, Hasler ED, Saxer S, et al. Effect of breathing oxygen-enriched air on exercise performance in patients with precapillary pulmonary hypertension: Randomized, sham-controlled cross-over trial. *Eur Heart J* 2017; 38: 1159–1168.

14. Van Campen JSJA, De Boer K, Van De Veerdonk MC, et al. Bisoprolol in idiopathic pulmonary arterial hypertension: An explorative study. *Eur Respir J* 2016; 48: 787–796.

15. Contini M, Apostolo A, Cattadori G, et al. Multiparametric comparison of CARvedilol, vs. NEbivolol, vs. BIsoprolol in moderate heart failure: The CARNEBI trial. *Int J Cardiol* 2013; 168: 2134–2140.

16. Dean JB, Mulkey DK, Henderson RA, et al. Hyperoxia, reactive oxygen species, and hyperventilation: Oxygen sensitivity of brain stem neurons. *Journal of Applied Physiology* 2004; 96: 784–791.

17. Guyenet PG, Bayliss DA. Neural Control of Breathing and CO2 Homeostasis. *Neuron* 2015; 87: 946–961.

18. Reybrouck T, Mertens L, Schulze-Neick I, et al. Ventilatory inefficiency for carbon dioxide during exercise in patients with pulmonary hypertension. *Clin Physiol* 1998; 18: 337–344.

Table 1 – Effects of bisoprolol and hyperoxic breathing on ventilation at rest and during exercise. *AT = anaerobic threshold, HR = heart rate, VE = total minute ventilation, RR = respiratory rate, VT = tidal volume,* $P_{ET}CO_2$ *= end-tidal partial pressure of* $CO_2$*,* $SaO_2$ *= oxygen saturation,* $FiO_2$ *= inspired fraction of oxygen.*

| | (Mean ± SD) | Baseline (n=17) | Placebo (n=16) | Bisoprolol (n=16) | Effect of bisoprolol (p-value) | Effect of hyperoxia (p-value) | Interaction bisoprolol*hyperoxia (p-value) |
|---|---|---|---|---|---|---|---|
| **CPET** | **Work (watt)** | 66±30 | 59±34 | 67±31 | *0.2914* | | |
| | **$VO_2AT$ (l/min)** | 0.70±0.20 | 0.64±0.24 | 0.65±0.20 | *0.5194* | | |
| | **$VO_2max$ (l/min)** | 1.01±0.34 | 0.95±0.41 | 0.98±0.34 | *0.5200* | | |
| | **$VO_2$/kg (ml/kg/min)** | 15.2±4.6 | 13.8±5.2 | 14.6±4.5 | *0.9337* | | |
| | **HR start (bpm)** | 80±14 | 76±13 | 74±18 | *0.8204* | | |
| | **HR max (bpm)** | 138±21 | 124±26 | 123±27 | *0.9279* | | |
| | **$O_2$pulse (ml/beat)** | 7.2±1.7 | 7.6±2.7 | 7.9±2.1 | *0.6477* | | |
| | **$VE_{peak}$ (l/min)** | 54.2±24.0 | 48.1±28.0 | 49.4±16.7 | *0.9444* | | |
| | **$RR_{peak}$ ($min^{-1}$)** | 38±18 | 35±20 | 36±10 | *0.8462* | | |
| | **$VT_{peak}$ (l)** | 1.4±0.4 | 1.4±0.4 | 1.4±0.3 | *0.2105* | | |
| | **$VE/VCO_2$ slope** | 42.4±11.0 | 42.1±11.5 | 39.8±11.0 | *0.8425* | | |
| **ABG** | **$PaCO_2$ (mmHg)** | 32.3±4.7 | 32.2±0.8 | 32.8±3.1 | *0.5737* | | |
| | **$PaO_2$ (mmHg)** | 73.5±21.5 | 67.2±13.0 | 71.3±15.8 | *>0.9999* | | |
| **Vent $FiO_2$ 21%** | **VT (l)** | 0.8±0.2 | 0.7±0.2 | 0.8±0.2 | *0.0149** | | |
| | **VE (l/min)** | 11.0±3.0 | 10.9±2.1 | 10.7±2.8 | *0.1751* | | |
| | **RR (breaths/ min)** | 14.5±3.1 | 15.8±3.0 | 15.1±4.1 | *0.2784* | | |
| | **$P_{ET}CO_2$ (kPa)** | 3.6±0.6 | 3.7±0.6 | 3.7±0.4 | *0.4822* | | |
| | **SaO2 (%)** | 93.0±5.7 | 92.4±5.2 | 92.0±7.0 | *0.3458* | | |
| | **HR (bpm)** | 78±12 | 78±13 | 66±13 | *<0.0001** | | |
| **Vent $FiO_2$ 40%** | **VT (l)** | 0.8±0.2 | 0.8±0.2 | 0.8±0.2 | *0.0149** | *0.4726* | *0.1634* |
| | **VE (l/min)** | 11.2±2.7 | 11.0±2.4 | 11.3±2.6 | *0.1751* | *0.2742* | *0.1518* |
| | **RR (breaths/ min)** | 14.7±3.4 | 16.3±2.9 | 15.4±4.2 | *0.2784* | *0.6032* | *0.4689* |
| | **$P_{ET}CO_2$ (kPa)** | 3.6±0.6 | 3.7±0.6 | 3.7±0.5 | *0.4822* | *0.2418* | *0.7034* |
| | **SaO2 (%)** | 96.9±4.4 | 96.1±4.9 | 96.1±6.4 | *0.3458* | *0.0012** | *0.8132* |
| | **HR (bpm)** | 74±12 | 75±13 | 64±13 | *<0.0001** | *0.0044** | *0.2906* |
