# Supplementary material for: Bisoprolol and/or hyperoxic breathing do not reduce hyperventilation in pulmonary arterial hypertension patients
Source: Pulm Circ. 2021 Dec 1;11(4):20458940211057890. doi: 10.1177/20458940211057890 (PMC8671678; doi:10.1177/20458940211057890)
Supplement: Supplementary file 1 — Supplementary Material [file PUL2-11-20458940211057890-s002.pdf]

Point-by-point response to the reviewers' comments

1. Please state whether the effects of long-term beta blocker treatment and or acute hyperoxic breathing on ventilation in patients with PAH or other disease(s) were ever evaluated in literature.

Thank you for this comment. We agree the background of what has been done already is rather limited, and added the following to lines 18-23:

It has been shown previously that 4 months of carvedilol treatment improves ventilatory efficiency in chronic heart failure patients [12]*. In addition, patients taking beta blockers showed to have lower minute ventilation (VE) both at rest and during exercise compared to patients not on beta blockers* [13]*. However, we are not aware of studies on the effect of long-term beta blocker use on ventilation in PAH patients. Yet, acute hyperoxic breathing increased ventilatory efficiency in PAH patients during, but not at the start of exercise* [14]*.*

2. line 5: "…and rest…" is "…at rest…"?

Thank you for pointing this out. We corrected it.
